# Monkeypox in Bulgaria: Significance of Various Clinical Samples, Clinical Manifestation, and Molecular Detection

**DOI:** 10.3390/jcm13164856

**Published:** 2024-08-17

**Authors:** Stefka Krumova, Radostina Stefanova, Petia Genova-Kalou, Daniel Ivanov, Maria Pishmisheva, Stanislav Kotsev, Iva Christova

**Affiliations:** 1 National Reference Laboratory “Measles, Mumps, Rubella”, Department of Virology, National Center of Infectious and Parasitic Diseases, 1233 Sofia, Bulgaria; rss_94@abv.bg (R.S.); iva_christova@yahoo.com (I.C.); 2 National Reference Laboratory of Cell Cultures, Rickettsia and Oncogenic Viruses, Department of Virology, National Center of Infectious and Parasitic Diseases,1233 Sofia, Bulgaria; petia.d.genova@abv.bg; 3 University Hospital for Infectious and Parasitic Diseases “Prof. Iv. Kirov”, 1000 Sofia, Bulgaria; dannieltiv@gmail.com; 4 MBAL “Pazardzhik”, 4400 Pazardzhik, Bulgaria; pishmishevampeleva@abv.bg (M.P.); kotsevstanislav@gmail.com (S.K.)

**Keywords:** monkeypox virus, orthopoxviruses, real-time PCR

## Abstract

**Background/Objectives**: Monkeypox (mpox) is currently the most common orthopoxvirus (OPXV) zoonotic disease, and, since 2022, there has been atypical person-to-person transmission observed in non-endemic countries. The present study aimed to investigate the frequency of monkeypox virus (MPXV) and OPXV DNA detection in recommended and alternative clinical materials taken during the acute and convalescent phases of infection in Bulgarian patients. **Methods**: The study included laboratory investigation by real time PCR of 181 clinical samples from 42 Bulgarian patients with possible mpox infections. **Results**: MPXV DNA was detected in 23/181 (12.71%), and OPXV DNA in 20/181 (11.05%) clinical samples. There were six mpox-confirmed patients aged 23 to 44. At the highest frequency, MPXV and OPXV DNA were detected in samples of vesicular contents (6/6) and nasal/oropharyngeal secretions (5/6 and 4/6) during the first three days from the appearance of clinical symptoms. We demonstrated MPXV and OPXV DNA in alternative samples (urine, feces, ejaculate, and saliva), and in follow-up patient samples, taken two weeks after mpox confirmation in the convalescent phase (vesicular contentsand urine). **Conclusions**: Our findings suggested that MPXV may be detected in a larger set of clinical materials, including alternatives, where the virus can persist for more than two weeks.

## 1. Introduction

Monkeypox (mpox) is a zoonotic disease caused by the monkeypox virus (MPXV). MPXV represents a large double-stranded DNA virus belonging to the genus Orthopoxvirus (OPXV), family Poxviridae. Mpox is currently the most prevalent orthopoxvirus zoonosis in humans since the eradication of the variola virus [1,2,3].

MPXV was first isolated from monkeys and was identified as a human pathogen in the Democratic Republic of the Congo (DRC, formerly Zaire) in 1970 [4]. Over the last almost 50 years, human cases of mpox have been identified in 11 African countries, and MPXV is considered endemic in the DRC [1,5]. Based on serological data, MPXV is maintained by various mammalian species, with periodic introduction into human populations, where relatively short chains (≤7) of human-to-human transmission can occur [6,7]. Genetically, there are several clades of MPXV: Central African, clade I (covering the Congo Basin—Democratic Republic of Congo, Central African Republic, Republic of Congo, Gabon, and Cameroon), West African, clade IIa (covering Nigeria, Benin, Côte d’Ivoire, Liberia, and Sierra Leone) and 2022 MPXV, and clade IIb (the outbreak-causing monkeypox virus of 2022, which is phylogenetically distinct from prior clades I or IIa and with possible different virological properties) [8,9].

Since 2022, a multi-country outbreak of mpox has been reported, including in non-endemic countries, outside Africa. The first cases occurred in people who attended an international LGBT+ Pride event on the Spanish island of Gran Canaria, which was linked to transmission chains in several European countries [10,11]. The most affected WHO regions, ordered by the number of laboratory-confirmed cases, were the Region of the Americas, the African Region, the European Region, the South East Asia Region, and the Western Pacific Region. In 2022, the outbreak was concentrated in the countries of the European region, and in 2023, the regions that were mainly affected were the South East Asia Region and the Western Pacific Region [12]. As of 10 July 2024, 98,001 mpox cases had been identified in 118 countries and areas globally, of which only 7 countries had historically reported mpox [13].

However, locally acquired infections and community transmission had become predominant by the end of May 2022 in all affected countries [11]. The current outbreak is characterized by atypically rapid human-to-human transmission, with no reported animal reservoir, which is probably determined by the accumulation of genetic mutations in the viral genome, favoring the circulation of the virus in the public, especially among highly affected groups (such as men who have sex with men), as well as non-immune household members and healthcare workers [10,14]. The human-to-human transmission of MPXV can occur through respiratory secretions, direct contact, vertical transmission, percutaneous transmission, or indirect contact with fomites. People with mpox are considered infectious until all their lesions have crusted over, the scabs have fallen off, and a new layer of skin has formed underneath, usually taking 2 to 4 weeks [15]. It is also possible for the MPXV to persist for some time on clothing, bedding, towels, objects, electronics, and surfaces that a person with mpox has touched [16]. On the other hand, Accordini S. et al. [17] describe the possible transmission of the virus from patients with asymptomatic and unrecognized infections who are not initially confirmed with mpox. This virus-carrying may increase the chain of transmission through sexual contact or unusual routes [17].

In laboratory diagnostics for the virus, the main technique used is the nucleic acid amplification test (NAAT)–PCR identification of specific DNA sequences from the viral genome. The critical step in a reliable and accurate mpox diagnosis is choosing the correct clinical specimens from each patient for testing [18]. According to the WHO criteria, the recommended sample type for laboratory investigation is skin lesion material, including roofs from more than one lesion (e.g., lesion crusts) and swabs from lesion surfaces and/or exudates [19]. In addition to lesion samples, the collection of an oropharyngeal swab is also encouraged. Importantly, the data on the accuracy of this type of sample for mpox diagnosis are scarce, and therefore a negative throat swab sample should be interpreted with caution. The literature data regarding the use of other types of (alternative) samples for MPXV laboratory diagnosis are limited, and this is the subject of investigation and discussion in the present study.

According to the literature data and a WHO study, the Vaccinia-based smallpox vaccine can provide cross-protection against mpox in 80–85% of those immunized and can reduce infection severity in cases that do arise [20]. Although post-vaccination immunity declines with time, it is suggested that the variola vaccine should provide some degree of protection in adults over 50 years of age [21,22]. Given the official announcement by the WHO of the eradication of smallpox in the world in 1980, vaccination in Bulgaria and other countries was not carried out after that year. The last people vaccinated in Bulgaria were those born in 1976–1978; the vaccine was not administered to all children, and national smallpox immunity seroprevalence data cannot be found. An analysis of public databases from Taube et al. [20] shows that no smallpox vaccination coverage estimate could be found for 37% of countries and 14% of the world population. This makes a large part of the population vulnerable to orthopoxvirus infection and mpox. The presence of a scarification scar on the right hand (in the area of the deltoid muscle) should be considered a sure sign of a variola vaccination [21].

The work described here aimed to investigate the frequency of MPXV and OPXV DNA detection in recommended and alternative clinical materials taken during the acute and convalescent phases of infection in Bulgarian patients.

## 2. Materials and Methods

### 2.1. Patients

From May 2022 to April 2024, a total of 181 clinical samples from 42 patients (31 males and 11 females) aged 6 to 76 years with possible mpox infections were tested. The most patients were analyzed in 2022 (n = 21), followed by 2023 (n = 12), and 2024 (n = 9). All patients were screened and negative for viral infection with herpes simplex 1 (HSV 1) and 2 (HSV 2), enteroviruses, and measles. More than two clinical materials were collected and tested from each patient: samples of vesicle contents (n = 42), crust (n = 38), nasal/oropharyngeal swab (n = 42), urine (n = 16), feces (n = 18), ejaculate (n = 16), and saliva (n = 9) were taken in the first days from the onset of clinical symptoms. The vesicle contents and nasal/oropharyngeal swabs were placed and transported in viral transport media (VTM). The clinical samples were refrigerated (2 to 8 °C) or frozen (−20 °C) and transported to the laboratory within 48 h after collection, according to the requirements of the WHO (Laboratory Guidelines for the Detection and Diagnosis of Monkeypox Virus Infection—2 September 2022) [19]. The follow-up samples were requested from all patients confirmed for mpox one week after the first samples. Clinical specimens were tested at the National Reference Laboratory “Measles, Mumps, Rubella”, National Centre of Infectious and Parasitic Diseases, Sofia, Bulgaria.

### 2.2. PCR Assays

Viral DNA was extracted from all specimens using the PureLink Viral RNA/DNA Mini Kit (Thermo Fisherr Scientific Inc., Waltham, MA, USA). Screening for MPXV DNA (F3L gene) was performed using Monkeypox Virus Real-Time PCR kits (bioPerfectus Technologies Co., Shanghai, China) and for the OPXV DNA rpo18 gene using the SuperScript™ III Platinum™ One-Step qRT-PCR Kit (Thermo Fisher Scientific, Inc, Waltham, MA, USA) with the primers OPV rpo F1 and OPV rpo R1 and the probe OPV rpo MGB [23] (Table 1). The protocols were designed to detect both Central and West African clades. The PCR machine used was the Real-time PCR System Gentier 96E, Tianlong Technology Co., Xi’an, China.

The DNA template used for each PCR assay had a volume of 5 µL. Positive and negative controls were included in each real-time PCR reaction. According to the Centers for Disease Control and Prevention (CDC), the detection of human DNA (e.g., RNase P) was used for extraction control [24]. MPXV and OPXV DNA were detected on the FAM channel, and the Ct value ≤ 40 was interpreted as positive (+). According to the kit manufacturer and the literature, the protocols used do not cross-reactive with the following microorganisms: measles, varicella-zoster, Epstein–Barr, Herpes simplex virus 1 and 2, rubella, cytomegalovirus, and human herpesvirus 6, 7, and 8.

### 2.3. Statistical Analysis

For the statistical processing of the obtained data, the following statistical approaches were used:

- Determination of indicators of relative share (%) and confidence interval (95%CI), utilizing which the dependence of applied diagnostic approaches and used clinical materials were evaluated.
- Standard deviation (SD).

## 3. Results

Of all the tested patients, six (6/42, 14%, 95%CI 3.51 ÷ 24.49) were confirmed to have mpox infection. Regarding the clinical samples studied, MPXV DNA was detected in 23/181 (12.71%, 95%CI 7.88 ÷ 17.54), and OPXV DNA was detected in 20/181 (11.05%, 95%CI 6.48 ÷ 15.62) (Table 1). The calculated percentage of coincidence between the obtained results (positive or negative) in the detection of MPXV and OPXV DNA by the PCR protocols used in our group of patients was 98%. The statistical analysis between the two groups, MPXV DNA (+) and OPXV DNA (+), showed *p* = 0.459 and a statistical significance level of 54%. In 20 of the tested clinical samples from the six mpox-confirmed patients, both MPXV and OPXV DNA were detected. All confirmed patients were men who have sex with men, aged 23 to 44 (with an average age of 35.17 ± 7.91 years), and two of them were HIV-positive and on antiretroviral therapy. The commonest clinical features were fever (5/6, 83%) and vesicular–pustular rash in the perianal (6/6, 100%), penile (4/6, 67%), and limb (4/6, 67%) regions. One of the patients was hospitalized. Regarding the epidemiological data, three patients reported traveling abroad to Spain and the UK.

Among the examined clinical materials, MPXV DNA was found with the greatest frequency in specimens of vesicle contents and nasal/oropharyngeal swabs, in 6/6 and 5/6 confirmed patients, respectively. It was OPXV DNA that was detected in vesicle contents (6/6), nasal/oropharyngeal swabs (4/6), and urine (4/6). On the other hand, MPXV was less likely to be detected in samples from feces, only being observed in one patient (with 33 Ct). The highest viral concentration of MPXV and OPXV DNA was shown in specimens of vesicle contents and nasal/oropharyngeal swabs in the first three days of the rash (Table 2).

**Figure 1:**
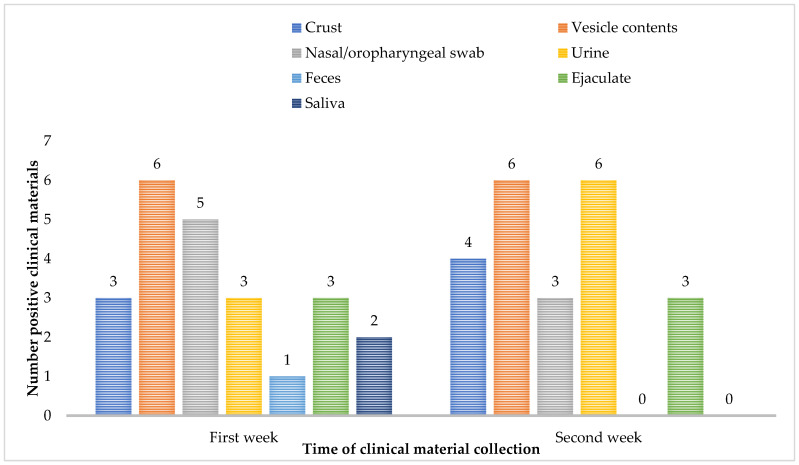
Evidence of MPXV DNA in the study of different types of clinical material samples taken during the acute and convalescent phase of infection in Bulgarian patients.

Follow-up samples were requested from all six confirmed patients, and MPXV DNA in the repeat samples was detected in the highest percentage in the specimen’s vesicle contents (6/6, 100%) and urine (6/6, 100%). In the second week after the confirmation of mpox infection, MPXV DNA was not detected in samples of feces and saliva (Figure 1).

## 4. Discussion

In the last multi-country mpox outbreak, MPXV was identified in non-endemic regions and people with no direct travel history, confirming a person-to-person transmission pattern in the community countries. Many surveys reported the new clinical and epidemiological pattern of the 2022 outbreak; the high frequency of lesions in anogenital areas and oral mucosa may explain transmission during sexual intercourse and not by animal reservoirs [25]. Clusters associated with sex-on-site venues, festivals, pride parades, and saunas have been reported, suggesting a correlation between mpox transmission and interconnected social networks [26].

A total of 27,180 mpox cases have been identified and reported in TESSy from 46 countries (including Bulgaria) and areas throughout the European Region up to 05 April 2024. The analysis of mpox cases shows that the majority of patients were male (98%) and between 31 and 40 years old, and 96% of the male cases with known sexual orientation self-identified as men who have sex with men. Among the cases with known HIV status, 38% were HIV-positive. Regarding clinical complications, 95% of cases presented with a rash and systemic symptoms such as fever, fatigue, muscle pain, chills, or headache (67%) [27]. Seven percent of mpox cases were hospitalized, and ten patients died. Globally, since 1 January 2022, cases of mpox have been reported to the WHO from 118 Member States across all six WHO regions [13].

All cases diagnosed in our cohort were young males and MSM, and some of them reported multiple sex partners. Transmission was overwhelmingly associated with person-to-person close contact and sexual activities, a recent history of traveling abroad (3/6, 50%), or contact with a confirmed mpox case (1/6, 17%). The proven epidemiological link determines infection with the virus outside the country’s borders.

The WHO recommends MPXV PCR testing primarily from skin lesions, which have higher sensitivity than other specimen types [28]; in our study, skin lesions also were the most common specimen type both during the first days from the onset of clinical symptoms and a week after. They were positive for MPXV and OPXV DNA between 26 and 30 Ct. In the first days of infection, MPXV DNA was demonstrated with high frequency in nasal/oropharyngeal swabs (5/6), and, in the later stages, in urine (6/6). This determined the passage of the virus through the host’s body—primary viremia in the upper respiratory tract and the subsequent excretion of virus particles through urine. Viral DNA was also shown in the ejaculate (3/6, 50%) and feces (1/6, 17%) samples from the patients, indicating the potential for the spread of the virus through sexual contact and household contact. Of course, the question remains as to whether the detected virus nucleic acid has infectious potential; this can only be proven by virus isolation in Vero cell cultures [29], and the present study was not carried out for this purpose. Our results showed that it is necessary for more than two types of clinical samples with the potential to detect the virus to be provided by each patient.

Since 2023, despite a decrease in the number of mpox cases in Europe, the main threat associated with the virus is the MPXV clade I outbreak in the Democratic Republic of Congo, which is endemic to Central Africa and is generally considered to be more virulent than MPXV clade II [30]. The previous mpox outbreak in the EU and worldwide was associated with the widespread circulation of clade II, and the population relies on cross-protection between the two clades. However, awareness among clinicians regarding the specificity of the virus and risk groups should be increased.

## 5. Conclusions

The main conclusions that we can draw from the present study are as follows:

In our practice, we successfully use different types of clinical material for MPXV and OPXV DNA detection. In addition to standard WHO-recommended samples (vesicle contents and nasal/oropharyngeal swabs), we demonstrated MPXV and OPXV DNA in alternative samples, namely, urine, feces, ejaculate, and saliva, including in follow-up patient samples, in which the virus can persist for more than two weeks. This supports screening a larger set of materials from a single patient and the easier transmission of the virus from person to person, including through household contact.

The concordance in 98% of the PCR results in proving MPXV and OPXV DNA shows the possibility of the parallel use of both methods for mpox detection.

Regarding the limitations of our study, we studied a small number of mpox-confirmed patients in Bulgaria, meaning that there is also a possible hidden spread of the virus without laboratory diagnosis; there could also be accumulations of mutations in the genetic regions that became undetectable with the primers and probe that we used. This study did not include analyses of alternative target genes (J2L/J2R genes, F2L/F3L genes, and the G2R gene) for MPXV detection. In the future, studies in this direction may improve mpox laboratory diagnostics.

Despite the known epidemiology and clinical manifestation of mpox, the human-to-human spread of the virus after May 2022 surprised many scientists and health authorities. This demonstrates the need for a more detailed investigation into virus transmission, genetics, and mutagenicity.

## Figures and Tables

**Table 1:** Real-time PCR assays for MPXV and OPXV diagnostics.

Pathogen	Target	Sequence (5′-3′)	Thermoprofile	Detection Limit *
MPXV	F3L gene	Specific primers and probes designed based on F3L gene areas of the monkeypox virus from bioPerfectus Technologies Co.	1 cycle: 95 °C for 300 s 45 cycles: 95 °C for 10 s 58 °C for 30 s	~5 copies of genomic DNA per reaction
OPXV	rpo18 gene	OPV rpo F1 CTgTAgTTATAAACgTTCCgTgTg OPV rpo R1 TTATCATACgCATTACCATTTCgA OPV rpo MGB FAM-CTgTAAACTAAgTAgAgATCC MGB	1 cycle: 95 °C for 300 s 40 cycles: 95 °C for 15 s 60 °C for 30 s	~10 copies of genomic DNA per reaction
Human DNA	RNase P	Forward: 5′-AGA TTT GGA CCT GCG AGC G-3′ Reverse: 5′-GAG CGG CTG TCT CCA CAA GT-3′ Probe: 5′-FAM-TTC TGA CCT GAA GGC TCT GCG CG-BHQ1-3′	1 cycle: 95 °C for 20 s 40 cycles: 95 °C for 3 s 60 °C for 30 s	-

* According to the manufacturer and the reference article [23].

**Table 2:** General information about the mpox-confirmed patients (n = 6).

Tested Clinical Specimens	Patient 1		Patient 2		Patient 3		Patient 4		Patient 5		Patient 6		Total MPXV (OPXV)
	Laboratory Results		Laboratory Results		Laboratory Results		Laboratory Results		Laboratory Results		Laboratory Results		
	MPXV PCR (Ct)	OPXV PCR (Ct)	MPXV PCR (Ct)	OPXV PCR (Ct)	MPXV PCR (Ct)	OPXV PCR (Ct)	MPXV PCR (Ct)	OPXV PCR (Ct)	MPXV PCR (Ct)	OPXV PCR (Ct)	MPXV PCR (Ct)	OPXV PCR (Ct)	
Crust	30 Ct	29 Ct	Neg	Neg	31 Ct	Neg	32 Ct	30 Ct	Neg	Neg	Neg	Neg	3 (2)
Vesicle contents	28 Ct	29 Ct	27 Ct	29 Ct	28 Ct	26 Ct	29 Ct	28 Ct	28 Ct	29 Ct	29 Ct	30 Ct	6 (6)
Nasal/oropharyngeal swab	Neg	Neg	25 Ct	Neg	27 Ct	28 Ct	27 Ct	30 Ct	26 Ct	31 Ct	28 Ct	29 Ct	5 (4)
Urine	Neg	30 Ct	Neg	Neg	Neg	Neg	32 Ct	30 Ct	30 Ct	32 Ct	33 Ct	31 Ct	3 (4)
Feces	Neg	Neg	Neg	Neg	Neg	Neg	33 Ct	Neg	Neg	Neg	Neg	Neg	1 (0)
Ejaculate	31 Ct	30 Ct	Neg	Neg	31 Ct	Neg	30 Ct	31 Ct	Neg	Neg	Neg	Neg	3 (2)
Saliva	Neg	Neg	Neg	Neg	Neg	Neg	29 Ct	30 Ct	Neg	Neg	26 Ct	29 Ct	2 (2)
Total number of positive specimens	3	4	2	1	4	2	7	6	3	3	4	4	23 (20)

## Data Availability

The data presented in this study are available on request from the corresponding author due to ethical reasons.
